# Musculoskeletal miRNA profiling after exposure to simulated space stressors in mice

**DOI:** 10.17912/micropub.biology.001745

**Published:** 2025-10-08

**Authors:** Carly Orr, Emma Mackey, Azemat Jamshidi-Parsian, Stephanie Byrum, Rupak Pathak, Robert Griffin, Nathan S. Reyna

**Affiliations:** 1 Biology, Ouachita Baptist University, Arkadelphia, Arkansas, United States; 2 University of Arkansas for Medical Sciences, Little Rock, Arkansas, United States; 3 Center for Proteomics and Metabolomics, St. Jude Children's Research Hospital, Memphis, Tennessee, United States; 4 Division of Radiation Health, University of Arkansas for Medical Sciences, Little Rock, Arkansas, United States; 5 Department of Pharmaceutical Sciences, University of Arkansas for Medical Sciences, Little Rock, Arkansas, United States

## Abstract

Prolonged spaceflight exposes astronauts to chronic irradiation and microgravity, inducing oxidative stress through the production of reactive oxygen species (ROS). This study identified two microRNAs, Mir6236 and Mir6240, that were significantly downregulated in murine skeletal muscle following simulated space conditions. Sequencing and bioinformatics analysis revealed these microRNAs likely regulate key ROS-associated genes and pathways, including FN1, EZR, TRX2, and MAP2K1. Their dysregulation suggests a role in tumor progression, underscoring the need to further investigate microRNA-mediated gene regulation under space-like conditions to better understand the long-term health risks associated with extended space travel.

**Figure 1. Gene expression analysis of Mir6236 and Mir6240 in mice raised in simulated space conditions:**
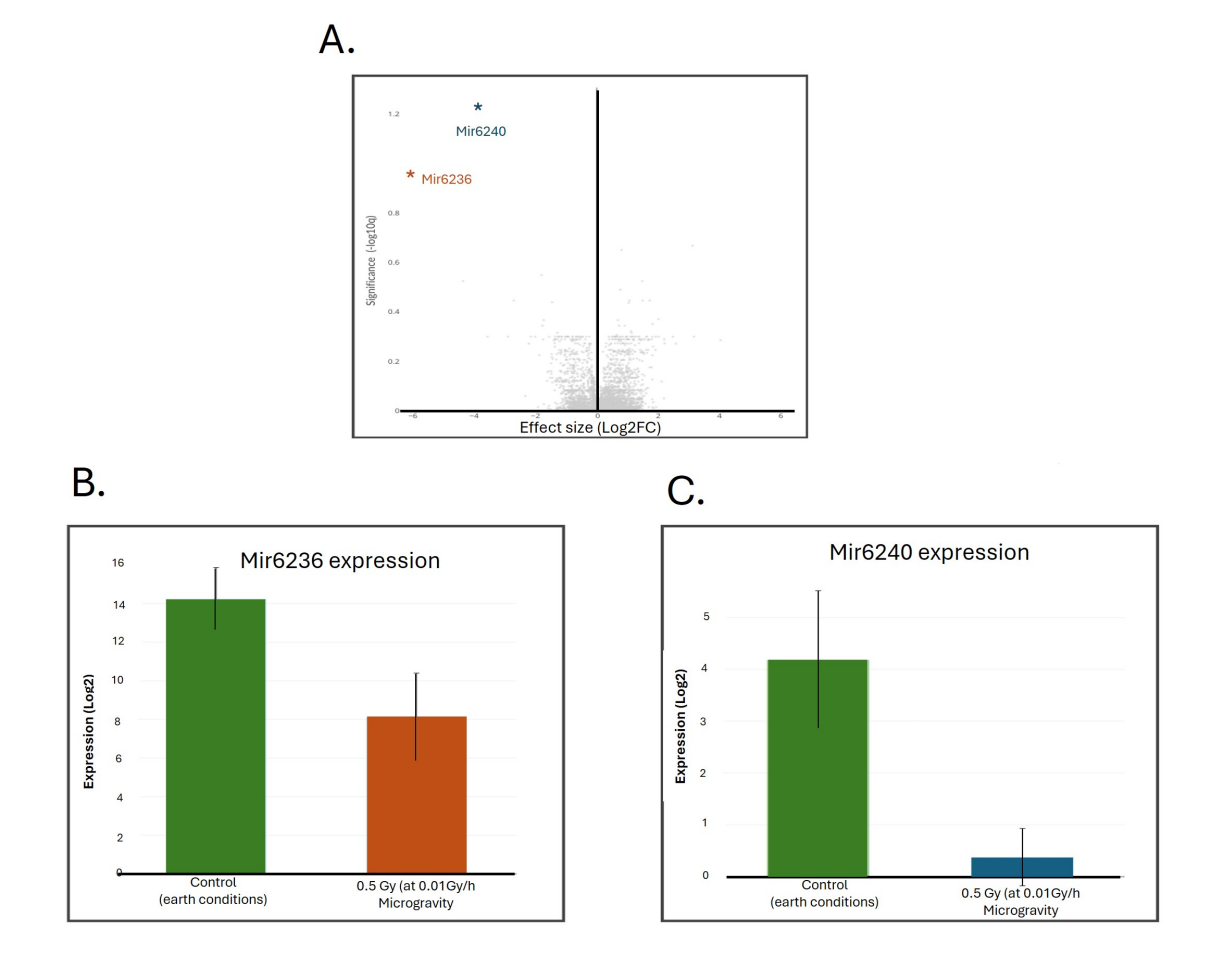
(A) Volcano plot of differentially expressed genes after exposure to simulated space conditions. (B) Bargraph showing the LFC of the expression of Mir6236 between the control and treatment (simulated space conditions) groups. (C) Bargraph showing the LFC of the expression of Mir6240 between the control and treatment (simulated space conditions) groups.

## Description

Oxidative stress presents a significant challenge for astronauts, as prolonged exposure to microgravity and space radiation leads to increased production of reactive oxygen species (ROS), resulting in cellular damage, DNA mutations, accelerated aging, and an elevated risk of cancer and neurodegenerative diseases (Azzam et al., 2011; Steller et al., 2018). The body mitigates the effects of ROS through regulatory pathways such as the WNT and MAPK pathways (Iqbal et al., 2024). These ROS-associated pathways are modulated by various factors, including microRNAs—small RNA fragments that bind to messenger RNAs (mRNA) and alter gene expression (O’brien et al., 2018). However, many microRNAs remain poorly annotated, and their specific functions are largely unknown (Fromm et al., 2022). This study identified two such microRNAs, Mir6236 and Mir6240, as significantly relevant and investigated their potential functions. MiR-6240 and miR-6236 are emerging as essential regulators in cancer research due to their predicted roles in modulating pathways associated with oxidative stress and reactive oxygen species (ROS) (Zhang et al., 2021). Dysregulation of ROS is a hallmark of many cancers, contributing to genomic instability, tumor progression, and resistance to therapy (Selvaraj et al., 2025). Understanding how miR-6240 and miR-6236 regulate ROS-related pathways could reveal novel therapeutic intervention targets and clarify tumor development mechanisms in response to oxidative stress.

To investigate the effects of chronic irradiation and simulated microgravity on the microRNA profile, C57BL/6J mice were housed in a space simulation facility at the University of Arkansas for Medical Sciences (UAMS). For 30 days, the mice were exposed to prolonged Cs-137 gamma irradiation. A NASA-approved hind-limb uploading model was used to simulate microgravity. Experimental mice were moved back to Earth gravity with no radiation for an additional 60-day growth phase, allowing us to assess the late impact of exposure. Control mice were raised in the same facility but under Earth gravity without irradiation. After the experiment, all mice were euthanized, and skeletal muscle tissue[RP1] was harvested for RNA sequencing.

Raw sequencing reads were analyzed using BigOmics Analytics RNA-Seq platform (BigOmics Analytics LLC, Sweden) for analysis. BigOmics is a graphical interface (GUI) that allows users to conduct a wide range of bioinformatics analyses (Akhmedov et al., 2020). First, sample quality control and principal component analysis (PCA) were used to determine data quality and structure.

The challenging nature of simulating space flight (microgravity/gamma radiation) limited our sample number and power to detect differences in gene expression. To provide a more reliable differential gene expression analysis, mRNA expression levels in control vs experimental mice were determined using three separate methods: DESeq2 (Love, Huber, and Anders, 2014), edgeR (Robinson, McCarthy, and Smyth, 2010), and limma (Ritchie et al., 2015). A volcano plot analysis ( Figure 1A ) revealed Mir6240 and Mir6236 as the only two microRNAs differentially expressed at a statistically significant level (p < 0.05) across all three statistical methods.

Mir6236 exhibited a -6.110 Log _2_ Fold Change (LFC), indicating its downregulation in the treatment group ( Figure 1B ). A meta-analysis of previously sequenced data by Zhou et al. (2020) found that Mir626 likely targets and downregulates the FN1 gene which encodes fibronectin -- a high-molecular-weight glycoprotein that plays a crucial role in cell adhesion, migration, growth, and wound healing (Speziale, Arciola, and Pietrocola, 2019). The downregulation of FN1 triggers the downregulation of HCLS1 (Zhou et al., 2020). The end result is an increase in cell migration and invasion, ultimately contributing to proteoglycan formation and tumor progression (Caponnetto et al., 2020). Analysis of the sequenced data from this study revealed that the FN1 gene had a LFC of 3.110 and HCLS1 gene had an LFC of 0.5389, values higher than most of the genome, suggesting these genes are likely targets of downregulated Mir6236. These findings align with existing literature, as FN1 and HCLS1are linked to ROS buildup[RP2] . (Martins et al., 2021; Kim et al., 2022; Qureshi-Baig et al., 2019). Because ROS buildup from prolonged low-dose radiation raises cancer risk (Cohen, 2007) and Mir6236 downregulation appears to affect ROS-related genes, Mir6236 likely plays a key role in tumor progression by modulating oxidative stress response pathways.

Further analysis of differential expression bar graphs revealed that Mir6240 exhibited an LFC of -3.802, indicating downregulation after treatment ( Figure 1C ). Mir6240 belongs to the mir-330-3p microRNA family (TargetScanMouse, release 8.0). The human analog of Mir6240, mir-330-3p, has been found to negatively regulate Thioredoxin 2 (TRX2), a gene that, when properly regulated, inhibits tumor progression but, when upregulated, enhances tumor metastasis and viability (Yao, Zuo, and Wei, 2018; Wang et al., 2020). Sequencing data showed TRX2 had an LFC of 0.2387, higher than most genomic LFC values, suggesting it as a potential Mir6240 target.

TRX2 is part of the thioredoxin (TRX) family of mitochondrial proteins that scavenge ROS produced in the cell (Chen et al., 2016; Bu et al., 2017). TRX2 is generally expressed in tissues with high metabolic demand, such as muscle tissue (Huang et al., 2016). The ratio of reduced to oxidized TRX2 is responsible for the activation of the protein apoptosis signal-regulating kinase 1 (ASK1). Because prolonged radiation causes ROS buildup that can lead to cellular damage and cancers like melanoma (Fink and Bates, 2005; Srinivas et al., 2019), Mir6240 likely plays a key role in tumor progression by modulating genes in oxidative stress response pathways and may cause unexpected health risks associated with extended space flights.

Additional literature shows that mir-330-3p negatively regulates the MAPK pathway through targets like MAP2K1 and GRIA3, both upregulated in the sequenced skeletal muscle tissue (Jin et al., 2019; Safa et al., 2020). This study’s sequencing data showed that the MAP2K1 gene had an LFC of 0.2688, while the GRIA3 gene had an LFC of 0.4153—values higher than those observed in most of the genome. MAPK transcripts were generally upregulated, as expected with the observed downregulation of Mir6240 (mir-330-3p). This aligns with prior research, as MAP2K1 and GRIA3 are key MAPK pathway components involved in mitigating ROS-related damage (Jin et al., 2019; Wei et al., 2020). Thus, Mir6240 likely regulates cellular damage and proliferation by targeting oxidative stress response genes, including TRX2 and MAPK pathway components MAP2K1 and GRIA3. The dysregulation of oxidative stress defense and tumor suppression pathways underscores the need to investigate microRNA-mediated gene regulation under space-like conditions further.

## Methods

**Hindlimb unloading (simulated microgravity) and Gamma Radiation**

Six-month-old male and female C57BL/6J mice were housed in a 175 cm² trapezoidal cages equipped with hind-limb unloading (HLU) mechanisms. Hind limbs were suspended at a 30° angle using a tail harness attached to a swivel buckle mounted to a guide wire, allowing full access to all areas of the cage (Globus and Morey-Holton, 1985; Morey-Holton and Globus, 1998). Cages were arranged at equal distances in a circular formation around a Cs-137 gamma source. After 30 days, the treatment mice received a cumulative dose of 0.5 Gy at a rate of 0.01 Gy/h. After 30 days of exposure (radiation/microgravity), mice were returned to their cages and housed without radiation or microgravity for an additional 60 days before tissue harvest. Control mice were raised in the same facility without hind-limb unloading and shielded from gamma radiation. All animal experiments were approved by the IACUC Committee of the University of Arkansas for Medical Sciences (IACUC- No. 4139), and all procedures were conducted in accordance with relevant guidelines and regulations.

**Tissue Harvest**

Immediately after the animals were euthanized, skeletal muscle tissue was harvested using sterile microsurgical tools and cut into ≤0.5 cm pieces, and each piece was placed in 1 ml of *RNAlater* Stabilization Solution according to the manufacturer’s recommendations (Invitrogen, catalog #AM7020). Samples in RNAlater were transferred to a 4°C refrigerator for 2 days to allow thorough penetration of the solution into the tissue. After 2 days, excess RNAlater solution was removed, and the samples were stored at -80°C until processing for the RNA preparation and sequencing

**RNA Isolation**

Frozen tissue was homogenized in ZR BashingBead Lysis Tubes (Zymo, catalog # S6003) using Bullet Blender 5E Gold (Next Advance). Total RNA was extracted from the homogenized tissue using Quick-DNA/RNA ^TM^ Miniprep Plus Kit (Zymo Research, catalog # D7003) as described in the manufacturer’s instructions. The final RNA concentration was measured by Qubit™ RNA BR Assay (Thermo Fisher Scientific, catalog # Q10210). RNA quality was assessed by Fragment Analyzer System (Agilent) using RNA kit (15 nt) (Agilent, catalog # 5191-6572) ( *Schroeder A et al., 2006* ). DV200 values representing the percentage of RNA fragments above 200 bp in length were determined as described previously ( *Matsubara et al., 2020* )

**RNA Sequencing** .

1 µg of total RNA was used as input to generate sequencing libraries using TrueSeq Stranded Total RNA Library Prep Gold Kit (Illumina, catalog # 20020599) following the manufacturer’s protocol in the reference guide. Briefly, total RNA was rRNA-depleted, fragmented, and reverse transcribed into cDNA. Then, double-stranded cDNA libraries were prepared by A-tailing adaptor ligation and index PCR amplification. The concentration of final libraries was assessed by Qubit™ 1X dsDNA HS Assay (Thermo Fisher Scientific, catalog # Q33231). The quality control of libraries was performed on the Fragment Analyzer System (Agilent) using HS NGS Fragment kit (1-6000 bp) (Agilent, catalog # 5191-6578), and QuantStudio Real-Time PCR system using KAPA Library Quantification Kit (Kapa Biosystems, catalog # KK4824). Libraries were sequenced either on a NextSeq2000 or NovaSeq 6000 (Illumina) with paired-end mode (read1: 101 cycles, read 2: 101 cycles, i7: 8 cycles, i5: 8 cycles)

**RNA-sequencing Bioinformatics Analysis**

RNA reads were checked for quality of sequencing using FastQC, v0.11.8. The adaptors and low-quality bases (Q < 20) were trimmed to a minimum of 36 base pairs using Trimmomatic, v0.39 (Bolger, Lohse and Usadel, 2014). Reads that passed quality control were aligned to the reference genome, *Mus musculus* GRCm39.104, using STAR, version 2.7.1a (Dobin et al., 2013). Raw counts were obtained from BAM files using Subread's features Count function (vs 2.0.0).

RNA sequencing (RNA-seq) count data were analyzed using the BigOmics Analytics platform, a web-based bioinformatics tool designed for interactive data exploration and differential expression analysis. The raw RNA-seq counts underwent filtering to remove genes with low expression. Genes were excluded if they did not meet a minimum count threshold across a sufficient number of samples. To account for differences in sequencing depth and RNA composition across samples, the data were normalized using the method appropriate for each statistical framework. The counts were normalized based on the median-of-ratios for DESeq2, edgeR applied the trimmed mean of M-values (TMM) normalization method, and limma used the vroom transformation to model the mean-variance relationship and produce precision weights for linear modeling.

Differential expression was assessed using three parallel statistical frameworks to ensure robust and cross-validated results. DESeq2 is a model based on a negative binomial distribution fitted to the count data, where dispersion estimates are moderated across genes, and the likelihood ratio test was applied. edgeR relies on negative binomial modeling with empirical Bayes moderation of dispersion estimates. Exact tests were used for pairwise comparisons. Limma-voom employed linear models fitted to the log-counts per million (log-CPM) data. Empirical Bayes moderation is also applied to improve the variance estimates across genes. Each method provided log2 fold change estimates and associated p-values, which were adjusted for multiple testing using the Benjamini-Hochberg false discovery rate (FDR). Genes with FDR-adjusted p-value < 0.05 and absolute fold change > 2 were considered significant.

BigOmics Analytics provided interactive visualizations, including principal component analysis (PCA) and volcano plots, to facilitate quality control and interpretation of results. The use of multiple statistical methods enabled users to cross-validate the results.
